# Proteomic Investigations into Hemodialysis Therapy

**DOI:** 10.3390/ijms161226189

**Published:** 2015-12-10

**Authors:** Mario Bonomini, Vittorio Sirolli, Luisa Pieroni, Paolo Felaco, Luigi Amoroso, Andrea Urbani

**Affiliations:** 1 Nephrology and Dialysis Institute, Department of Medicine, G. d’Annunzio University, Chieti-Pescara, SS. Annunziata Hospital, Via dei Vestini, 66013 Chieti, Italy; vsirolli@unich.it (V.S.); p.felaco@gmail.com (P.F.); lamoroso@unich.it (L.A.); 2 Proteomics and Metabonomics Laboratory, Istituto di Ricovero e Cura a Carattere Scientifico (IRCCS) S. Lucia Foundation, 00179 Rome, Italy; luisa.pieroni@uniroma2.it (L.P.); andrea.urbani@uniroma2.it (A.U.); 3 Department of Surgery and Experimental Medicine, Tor Vergata University, 00134 Rome, Italy

**Keywords:** proteomic, hemodialysis, uremic toxins, biocompatibility, dialysis membrane

## Abstract

The retention of a number of solutes that may cause adverse biochemical/biological effects, called uremic toxins, characterizes uremic syndrome. Uremia therapy is based on renal replacement therapy, hemodialysis being the most commonly used modality. The membrane contained in the hemodialyzer represents the ultimate determinant of the success and quality of hemodialysis therapy. Membrane’s performance can be evaluated in terms of removal efficiency for unwanted solutes and excess fluid, and minimization of negative interactions between the membrane material and blood components that define the membrane’s bio(in)compatibility. Given the high concentration of plasma proteins and the complexity of structural functional relationships of this class of molecules, the performance of a membrane is highly influenced by its interaction with the plasma protein repertoire. Proteomic investigations have been increasingly applied to describe the protein uremic milieu, to compare the blood purification efficiency of different dialyzer membranes or different extracorporeal techniques, and to evaluate the adsorption of plasma proteins onto hemodialysis membranes. In this article, we aim to highlight investigations in the hemodialysis setting making use of recent developments in proteomic technologies. Examples are presented of why proteomics may be helpful to nephrology and may possibly affect future directions in renal research.

## 1. Introduction

Uremia is a clinical syndrome resembling systemic poisoning [1], characterized by a variety of clinical symptoms that develop and worsen as kidney failure proceeds, due to the retention of various solutes, which are normally excreted by the kidney, called uremic toxins. The principal aim of renal replacement therapies is the removal of uremic toxins, targeted at an improvement in quality of life and survival. Hemodialysis (HD) is by far the most commonly used modality for chronic renal replacement: more than 1.7 million patients are currently treated with HD worldwide, a number that is growing at a rate of approximately six-to-seven percent annually.

In the extracorporeal HD system, blood is allowed to flow via a peristaltic pump into a special filter (hemodialyzer) whereby waste products and excess water are removed across a semipermeable membrane separating flowing blood from the dialysate stream; the cleaned blood is then returned to the patient’s body, while wastes are discharged. The main determinant of the success and the quality of HD therapy is represented by the artificial membrane packed into the hemodialyzers. Membranes are thin barriers capable of providing the removal of substances between adjacent phases, so that chemical and biophysical control consistent with continued survival is achieved. Moreover, protein adsorption following the contact of blood with the membrane material during the HD procedure is vital to the bio(in)compatibility of a membrane material, a justifiable concern in dialysis [2,3].

**Figure 1:**
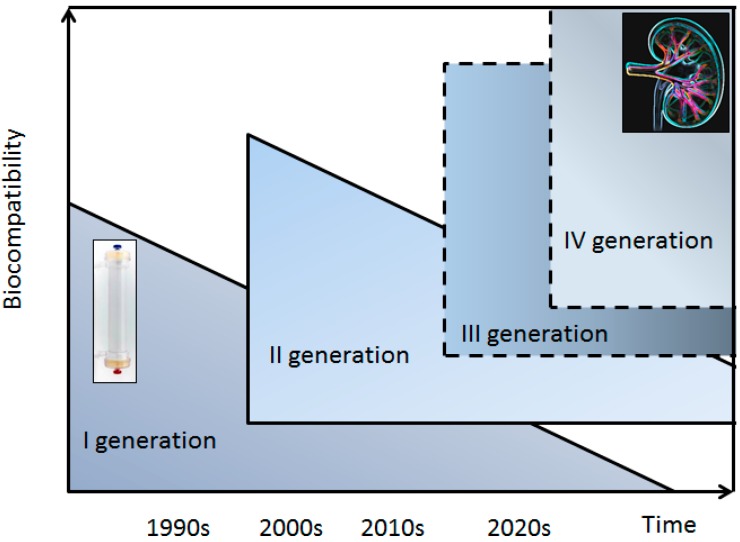
Evolution over time of biomaterials for hemodialysis membrane. Solid lines represent the material generation currently available in Hemodialysis (HD), dashed lines are used for material generation which are not presently available.

Today, most membrane materials follow the concept of first generation biocompatible materials. In these biomaterials the engineering aims to achieve an appropriate combination of chemical and physical properties, which may be useful in replacing the basic function of the original tissue with a minimal response in the host [4]. Second generation materials, as defined by HD procedure associated with bioactive components in order to elicit a specific biological response at the interface of the material, are currently being developed by combining biochemically active compounds such as vitamin E to scavenge oxygen reactive species [5]. Although these materials provide a fundamental therapeutic technology for end-stage renal disease (ESRD) patients, they are still far from developing a precision healthcare approach dedicated to the specific physiopathological conditions of different individuals. As such, third and fourth generations biomaterials are not currently available in renal replacement therapies. To develop such membranes requires the collection of basic primitive systematic evidence that proteomic investigations may provide (Figure 1).

Proteomic investigations enable analysis of complex multivariate protein functional mechanisms in a defined biochemical experimental model, and can be performed either as unsupervised or as targeted analysis. Application of proteomics has become one of the leading technologies for increased understanding of the key role played by proteins and protein–protein interactions in all aspects of cell function. There is an increasing use of proteomic technologies for investigation into renal replacement therapy such as HD [6]. In the last 10 years, the application of 2 dimensional electrophoresis (DE) separation techniques has been almost completely substituted by the use of shotgun bottom-up liquid chromatography (LC)-mass spectrometry (MS)/MS approaches. These analyses are more convenient and reproducible, however they provide molecular information at the peptide level only, thus subtle definition of specific protein isoforms available in the 2DE is often missing. More recently, the definition of top-down strategies in high resolution LC-MS/MS experimental set up is providing a new ground to define the specific proteoforms and their tentative association with specific biological states.

These newly developed MS techniques have been successfully applied to research in uremic toxicity, with the discovery of novel uremic toxins and the potential to define a precise molecular approach to defining the biochemical nature of uremia. Proteomic investigations associate genomic information with functional insight into the mechanisms involved in the interactions between the artificial membrane material and blood, thus providing the basic knowledge for generation of third-generation HD biomaterials. Moreover, to develop new concepts in the engineering of smart-biomaterials—fourth generation materials that may mimic nature’s hierarchical structural assemblages providing a framework to underpin the spatial and temporal relationships of molecular events during the life span of a patient—will necessarily require the collection of proteomic data. In fact new multifactorial molecular evidence will be needed if we are to achieve the complexity necessary to mimic natural tissues.

In this article, we review the results of recent proteomic investigations in the setting of chronic HD therapy (Table 1). Studies of uremic solute removal tend to segregate into biological mechanisms, clinical associations and dialyzer kinetics. This review focuses on the latter.

**Table 1:** Major investigations of Proteomics in Hemodialysis Therapy.

Molecular definition of Uremic toxins Identification of prognostic biomarkers Blood purification efficiency testing Biocompatibility assessment of filter materials

## 2. Dialytic Solute Removal

Uremic toxins can be classified according to their physicochemical characteristics, which in turn affect how they are removed dialytically. Uremic retained solutes can thus be schematically classified [7] into three main groups: (1) the small water-soluble compounds (molecular weight (*M*_W_) < 500 Da), with urea as a prototype; (2) the larger “middle molecules” (*M*_W_ > 500 Da), with β_2_-microglobulin as a prototype; (3) the protein-bound compounds, which include both small and middle molecules, with *p*-cresol as a prototype. In 2003, The European Uremic Toxin Working Group (EUTox-group) classified 90 retention solutes; of these, 22 were middle molecules (12 having a *M*_W_ > 12 kDa), while 25 solutes, essentially small solutes, were protein bound [7]. Protein-bound uremic toxins may contribute to the pathophysiology of cardiorenal syndrome [8].

During the extracorporeal procedure, mechanisms removing unwanted solutes and excess fluid from blood include diffusion, convection, and/or adsorption [9]. Diffusion refers to the molecular movement of solutes from a region of greater concentration to a region of lesser concentration; it is particularly effective in removing small sized solutes into outflow dialysate fluid. The bulk movement of fluid in response to a hydraulic or osmotic force is referred to as convection, and results in the transport of small and medium sized solutes by the solvent drag into the ultrafiltrate. The importance of improving removal of middle molecules has been highlighted as a possible way to ameliorate the morbidity and mortality associated with HD [10]. High adsorptive properties of some hydrophobic synthetic membranes may also significantly contribute to solute clearance during the extracorporeal procedure. Noxious compounds that are removed by adsorption onto the HD membrane include β_2_-microglobulin, tumor necrosis factor, and peptides [11,12]. However, excessive protein adsorption can limit the diffusive and convective capacity of a membrane, thereby reducing its therapeutic usefulness. In addition, certain synthetic polymers can bind erythropoietin [13] and medications [14], resulting in unwanted effects.

Many factors influence the concentration of uremic solutes in dialyzed patients [15]. These factors may be patient related (age, body weight, food intake, and residual renal function), dialysis related (schedule, dialyzer characteristics, blood and dialysate flow), and solute related (*M*_W_, compartmental distribution, protein binding, electrostatic charge, and intracellular concentration). Compartmentalization of solutes has important implications, since removal is less affected by the use of high-flux or large pore size dialyzers for solutes that behave like large molecules due to multicompartmental distribution. The intermittency of HD treatment, which is generally provided thrice weekly, is another relevant factor contributing to fluctuations in solutes’ concentration. 

During the extracorporeal dialysis procedure, both intended and unintended removal may occur. Examples of the latter are represented by losses of water-soluble vitamins, and losses of proteins and amino acids, which are inevitably associated with a high removal of middle or large MW solutes. The concept of unintended removal is however closely linked to the lack of adequate knowledge about the toxicity of solutes. We clearly need a more specific removal of uremic toxins. However, to accomplish this, the basic metabolic disturbances taking place in uremia and the toxic compounds responsible for the disturbances have to be better defined.

## 3. Proteomic and Uremic Toxicity

To date, more than 115 uremic toxins have been identified [16], and more are expected. Two main points about uremic toxins and research into them emerged and have been highlighted in a recent paper [17]: (i) the importance of a standardized approach to testing the biologic effect of uremic retention solutes, using appropriate concentrations and control conditions, taking into account (especially for protein-bound solutes) the albumin content of the test medium, and excluding confounding factors like contamination by bacterial derivatives [18]; (ii) that the strength of the biological effect of uremic retention solutes is related to their concentration, which is affected not only by dialysis removal but also by endogenous metabolism generation, especially for the small water-soluble compounds such as the guanidine compounds or the purines, and the middle molecules [19,20]. Some molecules, like the advanced glycation end products (AGEs), are present in food and absorbed unmodified [19], whereas several protein-bound solutes and volatile compounds are metabolites produced by the natural digestion process [19,20,21,22], then transformed by the intestinal wall or the liver via conjugation [23]. For example, tyrosine is modified by the intestinal microbiota into *p*-cresol, to be further metabolized in the body to *p*-cresylsulfate and *p*-cresylglucuronide [24,25,26]. This further indicates the complexity of uremic toxicity and its biological/biochemical environment.

### 3.1. Characterization of Uremic Toxins

By using proteomic technologies, many efforts have been made to characterize uremic toxins [27]. In a pilot study of serum proteomic expression proteins in HD patients, surface-enhanced laser desorption/ionization time-of-flight MS (SELDI-TOF MS) analysis demonstrated 30 well-defined protein differences between patient and control samples, 15 proteins being increased and 15 proteins decreased; this similarity in protein profiles among HD patients suggests they have a major effect on patients’ physiological responses to ESRD and/or dialysis therapy [28]. In uremic ultrafiltrate obtained during HD with high-flux dialyzers, a total of 21 forms of six unique proteins were identified as potential uremic toxins [29]. A subsequent study by EUTox showed a low consensus between the polypeptide profile obtained during HD in both dialysate and ultrafiltrate and the urinary proteome profile, which suggests that renal replacement therapy cannot substitute all aspects of the kidney function, and that the artificial dialyzer membrane is not comparable with the native kidney [30,31]. Polypeptides > 5 kDa were almost exclusively detected in the uremic ultrafiltrate obtained with high-flux membranes [31]; two such polypeptides were identified as fragments of salivary proline-rich protein and α-fibrinogen [31], whose functional significance is, however, unclear. In a recent review of MS studies on uremic toxins, Niwa [32] highlighted how accumulation of protein-bound low-molecular weight solutes which are not efficiently removed by HD conventional membranes because of their high albumin-binding property, such as indoxyl sulfate, *p*-cresyl sulfate, and 3-carboxy-4-methyl-5-propyl-2-furanpropionic acid, might play an important role in the development of uremic complications. Because of the high levels of indoxyl sulfate in uremic patients and its proven uremic toxicity in experimental models [32], this molecule is considered the most promising protein-bound uremic toxin as a biomarker of progress in chronic kidney disease (CKD). More recently, plasma proteome of CKD stage 2–3 patients and CKD stage 5 patients on HD was examined using high-resolution LC-MS/MS analysis in a holistic, large scale, integrative approach [33]. Out of a total of 2054 detected proteins in the plasma of HD patients, 127 displayed lower, while 206 displayed higher abundance than the proteome of patients at an earlier CKD stage. Modification of known processes involved in CKD complications such as increased inflammation, complement activation and vascular damage, and decreased hemostasis was shown by molecular pathway analysis [33]. This was another effort to identify and characterize uremic toxicity, providing a comprehensive assessment of CKD plasma proteome.

### 3.2. Characterization of Protein Damage Products

Chronic uremia is characterized not only by an increase in oxidative stress, but also by a more generalized increase in “carbonyl stress” (carbonyl overload) resulting in chemical modifications of proteins and in accumulation of AGEs and advanced lipoxidation end products in plasma and tissue proteins [34]. Proteomic investigations have proved an effective tool for the identification and molecular characterization of protein damage products, a heterogeneous class of uremic toxins with pro-inflammatory and pro-oxidant properties [35]. In the uremic environment, proteins may undergo posttranslational modifications that are not detectable by standard analytical techniques and may render the protein toxic. A glycated form of β2-microglobulin, for example, is thought to be implicated in the occurrence of dialysis-associated amyloidosis [36] and in loss of membrane phospholipid asymmetry with phosphatidylserine exposure in human red blood cells [37], a finding of pathophysiological significance [38]. In addition, in uremic patients it has been demonstrated that plasma proteins are susceptible to carbonyl formation, an alteration including not only the major plasma proteins [39,40] but also several proteins present in the blood in small amounts [41]. Hemodialysis treatment may adversely affect the carbonyl balance and exacerbate carbonyl stress [41,42,43,44]. Identification of the exact biological mechanisms involved in HD-induced carbonylation of some plasma proteins might help define the dialysis strategies that best offset carbonyl overload. Indeed, although the pathophysiological significance of protein carbonylation in uremia remains to be definitively established, carbonyl stress may be of relevance to various long-term complications [45,46]. Carbonylation of fibrinogen may be involved to the impaired clotting activity found in patients on HD [47]; carbonylation of ceruloplasmin and haptoglobin can impair the antioxidant properties of those proteins [48,49]; accumulation of AGEs may be a pathogenetic factor for low bone turnover [50]; oxidative alteration of albumin may adversely affect its vasculoprotective effects [39,51]; carbonyl stress may contribute in the pathogenesis of alteration in left ventricular geometry and function [46]; and, finally, carbonylated albumin may play a role in the early atherogenic events of chronic uremia by directly damaging the endothelium [41].

### 3.3. Other Investigations

Some other investigations concentrate on the potential use of proteomics in exploring the uremic pathophysiology and in assessing the response to therapeutic interventions. Vitamin C deficiency is thought to be a contributing factor to sustained oxidative stress in HD patients [52]. Plasma proteomic assessment of HD patients receiving 250 mg oral vitamin C supplementation thrice weekly for two months revealed normalization of the abnormal levels of some polypeptides [53]. In a four-year prospective study on 134 HD patients, levels of 12 cytokines were measured using a proteomic biochip system and correlated to the clinical outcome [54]. A significant and specific enhanced hazard ratio of cardiovascular mortality (hazard ratio 11.32, 95% confidence interval 2.52 to 50.90; *p* < 0.01) was found to be associated with interleukin (IL) 6. Interestingly, the cytokine (IL-4 + IL-6 + IL-10)/(IL-2 + Interferon γ) ratio, a marker of lymphocyte T helper subset cytokine secretion, proved to be useful in identifying patients with an increased non cardiovascular mortality risk [54]. Furthermore, recent observations obtained by complementary proteomic approaches consistently indicate that the functional and compositional properties of high density lipoproteins (HDL) are affected by qualitative abnormalities in ESRD patients, which might impact on the proposed cardioprotective properties of HDL [55,56,57]. In particular, 22 proteins were significantly increased and six proteins significantly decreased [57]. Since protein increase in HDL from uremic blood has been associated with atherosclerosis and inflammation, quantification of proteins in HDL might be a useful tool when assessing the cardiovascular risk of HD patients: it should be noted that the cardioprotective mechanisms of HDL (cholesterol acceptor capacity and antioxidative capacity) remain suppressed after kidney transplantation, regardless of graft function [58].

## 4. Proteomic and Extracorporeal Blood Purification

Proteomics has also been used in the HD setting for critical assessment comparing the blood purification efficiency of different dialyzer membrane materials or different extracorporeal strategies, which can help in understanding the relevance of protein removal during renal replacement therapy.

### 4.1. Hemodialysis

A comparison between dialysate obtained from patients on high-flux and low-flux dialyzers by means of capillary electrophoresis coupled to MS showed 2515 different polypeptides (based on size and elution time) in the high-flux membrane filtrate and 1639 in the low-flux membrane filtrate [30]. Proteins removed into outflow dialysate fluid and proteins adsorbed onto two different dialysis membranes, polysulfone and polymethylmethacrylate (PMMA), were profiled during clinical HD by lshikawa *et al.* [59] using SELDI-TOF MS. A greater reduction rate of serum β_2_-microglobulin was found with the PMMA membrane. The intensity of the protein peak that SELDI identified as β_2_-microglobulin was greater in the outflow hemodialysate fluid when using the polysulfone membrane, whereas in contrast, the PMMA membrane adsorbed the protein in a greater amount. In a subsequent analysis, Dihazi *et al.* [60] detected in the dialysate fluid derived from high-flux membranes ≥ 58 SELDI peaks with a mass per charge of 2000–150,000, and in the dialysate derived from low-flux membranes only 6–10 spectra in the range of 2000–12,000. In the latter fluid, the absence of high *M*_W_ proteins was confirmed by western blot analysis [60]. The difference detected in the removal of uremic toxins reflects the lower efficacy of low-flux membranes. In a randomized cross-over study comparing three new-generation high-flux polysulfone membranes, sodium dodecyl sulfate-polyacrylamide gel electrophoresis (SDS-PAGE) (Bio-Rad Laboratories, Hercules, CA, USA) scanning of spent dialysate showed significant differences in the removal of middle molecules (MW 8–60 kDa), which were not detected by blood measurements. In particular, while no difference in removal of small molecular weight compounds was observed, β2-microglobulin reduction differed among the three dialyzers [61]. In a prospective cross-over study, we compared the plasma protein adsorption properties of cellulose triacetate, a modified cellulose membrane, and of the synthetic polymer polysulfone-based helixone [62]. Analysis of proteins in eluate using MS analysis (matrix-assisted laser desorption/ionization (MALDI)-TOF/TOF spectrometer, Bruker-Daltonics, Bremen, Germany), MS/MS sequencing and Shotgun analysis by nanoLC-MS demonstrated significant differences in the protein adsorption profile of the two membrane materials. We found in particular that 22 proteins tended to concentrate in helixone (low-abundance plasma proteins) and 32 in cellulose triacetate (mostly high-abundance plasma proteins) membranes, the difference being related to membrane material and not to patient characteristics [62].

### 4.2. Other Blood Purification Strategies

Pedrini *et al.* [63] employed Multidimensional Protein identification Technology to characterize in a prospective crossover study the removal of middle-molecular weight solutes during high-volume post-dilution hemodiafiltration (a mixed diffusive/convective dialytic technique) with two high-flux dialyzer membranes (Amembris and Polyamix). A total of 277 proteins were identified by the proteomic approach in the dialysate fluids, removal capability being higher with the Amembris membrane (B. Braun Avitum, Melsungen, Germany). This is another effort to steer research toward a better knowledge of uremic toxins and the balance between intended and unintended removal of undesired and beneficial proteins [63]. In a proteomic investigation on extracorporeal blood purification techniques, Monari *et al.* [64] showed that HFR (HemoFiltration with endogenous on-line Reinfusion), an integrated dialysis technique that combines convection, diffusion, and adsorption, is better than standard HD at removing uremic toxins, especially medium-high MW molecules. By analyzing the proteomic profile of ultrafiltrate samples collected during the extracorporeal procedure, the authors compared the extraction rate of two different polysulfone membranes used for the convective chamber in the HFR dialyzer: polyphenylene High Flux (pHF) and polyphenylene Super High Flux (pSHF). β_2_-microglobulin, cystatin C, complement factor D and A1AG1 (representative uremic retention solutes), NGAL (one of the most promising biomarkers of acute kidney injury and a stress marker in chronic dialysis), zinc-α_2_ glycoproteìn, transferrin and complement factor B (known to play a role in the inflammation process during kidney disease), were monitored. The investigation showed how in the initial phase (15 min) of the dialysis session, there is a higher extraction performance by pHF membrane (Bellco Srl, Mirandola, Italy), while conversely at the end of the treatment (235 min) all protein spots were being extracted to a greater extent by pSHF membrane (Bellco Srl). Overall, pSHF membrane displayed higher permeability and better performance in removing middle molecules related to uremic syndrome [64]. By using the same extracorporeal technique, Cuoghi *et al.* recently evaluated the performance of a new polysulfone membrane (Bellco Srl), Synclear 0.2, in removing uremic molecules [65]. This membrane was found to have higher purification efficiency than conventional ones. Use of Synclear 0.2 has enabled the limit of the “albumin wall” to be surmounted without loss of important nutrients, whilst clearing middle-high *M*_W_ uremic toxins, hence improving dialysis purification efficiency [65]. Besides convection, the results of a recent randomized cross-over study suggest that dialysis time extension may be another strategy to improve the removal of non-conventional uremic toxins [66]. The reduction ratio and total solute removal of protein-bound uremic toxins, AGEs and dicarbonyl compounds (as determined by ultra-performance LC-tandem MS) were calculated in 13 stable conventional HD patients randomly completing a single session of 4-h HD, 4-h hemodiafiltration (HDF), 8-h HD and 8-h HDF. Blood flow and dialysate flow were identical in HD and HDF treatments, thereby allowing one to separate the time and convection components. Extended HD and HDF proved to be associated with enhanced reduction ratio and removal of investigated compounds compared to shorter (4 h) sessions. The highest reduction ratio was obtained with extended HDF, though it was superior to extended HD for only a few compounds [66]. The results of this interesting study need to be investigated in future prospective trials to assess their clinical relevance.

## 5. Proteomics and Protein Adsorption onto Dialysis Membrane

An important feature of membranes used for chronic HD therapy in ESRD patients is represented by their adsorptive properties [67,68], largely depending on surface characteristics such as hydrophilicity, roughness, charge, and chemistry [69]. Deposition and adsorption of plasma proteins occurs almost immediately upon blood exposure to artificial membranes surfaces. In addition to the effects described in Section 2, protein adsorption mainly governs the bio(in)compatibility of membranes. Indeed, several biologic pathways such as the blood coagulation cascade, the complement and the fibrinolytic systems, and cellular mechanisms [70,71,72,73] may be triggered by the interaction with activated proteins surface-adsorbed, with potential pathophysiologic consequences. On the other hand, the biocompatibility profile of dialysis membranes might be increased by adsorption of complement fragments C3a and C5a [74], complement factor D [75], and high *M*_W_ kininogens [76]. Thus, during the development of biomaterials to be used for HD therapy protein adsorption should be carefully controlled [77], ideally by evaluating the amount, composition and conformational change of the proteins adsorbed. Hitherto, however, detailed analysis of protein adsorption onto HD membrane materials has been greatly limited by the absence of adequate protein separation and identification techniques.

Proteomic approaches may make it possible to investigate protein adsorption in an unbiased manner and in its full complexity. Over the last few years, we have been investigating the suitability of such techniques in the case of dialysis membranes. Our first *in vitro* HD system, using hollow-fiber minidialyzers, investigated the protein-binding characteristics of two different materials employed for HD membranes: cellulose diacetate and ethylenevinyl alcohol [78]. By two-dimensional PAGE and nanoLC-MS/MS analysis, we identified a number of different proteins as common or characteristic of filter material interaction. In particular, 10 proteins were adsorbed to a greater extent onto cellulose diacetate, whereas another four proteins were detected on ethylenevinyl alcohol membrane [78]. Subsequently, by developing an *ex vivo* apparatus to study protein adsorption in a parallel set of experiments, we investigated membrane biocompatibility by correlating the chemical feature of the membrane surface with the adsorbed proteome repertoire, the profiling strategy being based on 2DE separation of adsorbed proteins coupled to MALDI-TOF/TOF analysis [79]. Dialyzers composed of either cellulose triacetate or the polysulphone-based helixone materials were used. The total protein adsorption did not differ between the two materials investigated. However, while 179 protein spots were visualized for helixone a map of retained proteins onto cellulose triacetate was made up of 239 protein spots. In the cellulose triacetate map a number of different protein spots belonging to the gene transcript of albumin were identified. In contrast, helixone bound only a small proportion of albumin, but resulted particularly active in retaining proteins associated with the coagulation cascade such as the fibrinogen isoforms, and low molecular weight fragments of fibrinogen, which may reflect the occurrence of fibrinolytic and procoagulatory activity upon exposure of blood to the membrane material [79]. Our data suggest that proteomic techniques are a useful approach for the investigation of proteins surface-adsorbed onto HD membranes, and may also provide a molecular basis for interpreting the efficacy and safety of anticoagulation treatment used during renal replacement therapy to avoid clotting of the extracorporeal circuit. This issue was investigated more deeply in a recent investigation using shotgun proteomics (data-independent analysis) on eluate obtained with cellulose triacetate or helixone membrane materials after an *in vivo* dialysis clinical session [80]. The involvement of adsorbed proteins in important molecular processes such as lipid transport and metabolism, cell growth differentiation and communication, and the coagulation cascade was demonstrated by functional classification and network analysis performed using bioinformatics tools. The different panels of proteins adsorbed onto each membrane were further confirmed by targeted MS using selected reaction monitoring on proteotypic transitions of key protein effectors. A slightly higher platelet activation profile after the dialysis session using helixone filter cartridges also was found. These results highlight a modulation during HD of the coagulation profile, which is largely connected with the biomaterial used [80].

In other investigations, Ishikawa *et al.* [59] observed by SELDI-TOF MS analysis that 169 peptides were adsorbed more onto a PMMA membrane, while the polysulfone membrane adsorbed to a greater extent 68 peptides, particularly those with mass per charge < 11730. Aoike [81] reported a proteomic analysis in HD patients complaining of pruritus, a common and troublesome complication in such patients. It had been shown that use of a slightly anionic PMMA membrane (BG series) can ameliorate HD-associated pruritus [82]. By using two different proteomic approaches, a purification approach and a pre-filtration approach, it was observed that the slightly anionic PMMA membrane can adsorb compounds with a MW up to 160 kDa, which interestingly displayed a stimulating effect (histamine release) on mast cells [81]. Mares *et al.* [83] investigated the molecules involved in blood-dialyzer interaction during HD. Proteins adsorbed onto the polysulfone F6 low-flux membrane were eluted, separated by 2D-gel electrophoresis, and analyzed by tandem MS when present in all patients. Eighty-four out of 153 detected proteins were found in all patients, 57 of them being successfully identified as 38 components of 23 unique proteins. Ten protein spots with a relative eluate intensity significantly different from that in the plasma, which is suggestive of preferential adsorption and hence of a special interaction with the membrane, were revealed by quantitative intensity analysis and included ficolin-2 and clusterin, which are involved in complement activation. These data suggest a possible role by the lectin complement pathway upon contact of blood with the polysulfone membrane, which contributes to dialyzer bio(in)compatibility [83].

It thus appears that regardless of its origin, cellulosic or synthetic, each dialysis membrane has multiple and different characteristics and acquires a unique and characteristic protein layer following interactions with blood components. Identification of proteins surface-adsorbed onto HD membranes can give important insights into reactions taking place during the dialysis procedure and is therefore relevant to evaluating the material’s biocompatibility. The collected evidence supports the concept [84] that classification of membranes into biocompatible and bioincompatible, particularly with the advent of synthetically modified cellulose membranes, is not a simple division into synthetic and cellulosic membranes, respectively. In keeping with this, a recent study analyzing the dialysate proteome pattern showed strong interaction with plasma proteins resulting in significantly higher protein loss during dialysis sessions with the synthetic Polyflux membrane than with a modified cellulosic membrane [85].

## 6. Conclusions

Proteomic investigations are a powerful tool affording fundamental and advanced molecular knowledge in the field of uremia [86]. Further development of comprehensive molecular strategies such as proteomics will be essential for implementation of third and fourth generation biomaterials. Today, proteomics has allowed us to identify several previously unknown toxins, and to better characterize the removal profile of different HD dialyzers and systems. Proteomic analysis also proves a suitable approach to monitoring the adsorption of plasma proteins onto membranes used in HD therapy, the key factor determining the following events during the dialysis procedure. Despite the advances in mass spectrometry and protein technology, however, the translation of proteomics into new membrane materials and consequently into clinical practice is lagging behind. Membrane materials for HD are currently applying concepts that were introduced in other areas of biomaterials more than 25 years ago. Nevertheless, much current effort, including the development of improved omics-study designs, and multicenter validations with multidisciplinary teams possessing complementary knowledge, hold out promise of a more widespread and effective development of new materials.

Hemodialysis treatment should ideally ensure minimal activation of blood components upon blood–membrane material interaction and remove uremic-retained solutes in a defined MW range. Application of proteomics may provide information relevant to renal replacement therapy, and a valid support to manufacturers and dialysis technologists. This should lead to more biocompatible and efficient dialysis strategies, to the potential benefit of the uremic patient.

## References

[1] Vanholder R., van Laecke S., Glorieux G. (2008). The middle-molecule hypothesis 30 years after: Lost and rediscovered in the universe of uremic toxicity?. J. Nephrol..

[2] Bonomini V. (1991). Introducing the biology of bioincompatibility in dialysis. Nephrol. Dial. Transplant..

[3] Hakim R.M. (1993). Clinical implications of hemodialysis membrane biocompatibility. Kidney Int..

[4] Holzapfel B.M., Reichert J.C., Schantz J.T., Gbureck U., Rackwitz L., Nöth U., Jakob F., Rudert M., Groll J., Hutmacher D.W. (2013). How smart do biomaterials need to be? A translational science and clinical point of view. Adv. Drug Deliv. Rev..

[5] Yang S.K., Xiao L., Xu B., Xu X.X., Liu F.Y., Sun L. (2014). Effects of vitamin E-coated dialyzer on oxidative stress and inflammation status in hemodialysis patients: A systematic review and meta-analysis. Ren. Fail..

[6] Bonomini M., Sirolli V., Magni F., Urbani A. (2012). Proteomics and nephrology. J. Nephrol..

[7] Vanholder R., de Smet R., Glorieux G., Argilés A., Baurmeister U., Brunet P., Clark W., Cohen G., de Deyn P.P., Deppisch R. (2003). Review on uremic toxins: Classification, concentration and interindividual variability. Kidney Int..

[8] Lekawanvijit S., Krum H. (2015). Cardiorenal syndrome: Role of protein-bound uremic toxins. J. Ren. Nutr..

[9] Ronco C., Tetta C. (2007). Extracorporal blood purification: More than diffusion and convection. Does this help?. Curr. Opin. Crit. Care.

[10] Baurmeister U., Vienken J., Ward R.A. (2009). Should dialysis modalities be designed to remove specific uremic toxins?. Semin. Dial..

[11] Goldman M., Dhaene M., Vanherweghem J.L. (1987). Removal of β2-microglobulin by adsorption on dialysis membranes. Nephrol. Dial. Transplant..

[12] Bouman C.S., van Olden R.W., Stoutenbeek C.P. (1998). Cytokine filtration and adsorption during pre- and postdilution hemofiltration in four different membranes. Blood Purif..

[13] Cheung A.K., Hohnholt M., Leypoldt J.K., de Spain M. (1991). Hemodialysis membrane biocompatibility: The case of erythropoietin. Blood Purif..

[14] Rumpf K.W., Reiger J., Anjorg R., Doht B., Scheler F. (1977). Binding of antibiotics by dialysis membranes and its clinical relevance. Proc. Eur. Dial. Transplant. Assoc..

[15] Vanholder R., Ringoir S. (1992). Adequacy of dialysis: A critical analysis. Kidney Int..

[16] Vanholder R., Baurmeister U., Brunet P., Cohen G., Glorieux G., Jankowski J.A. (2008). Bench to bedside view of uremic toxins. J. Am. Soc. Nephrol..

[17] Vanholder R., Boelaert J., Glorieux G., Eloot S. (2015). New methods and technologies for measuring uremic toxins and quantifying dialysis adequacy. Semin. Dial..

[18] Cohen G., Glorieux G., Thornalley P., Schepers E., Meert N., Jankowski J., Jankowski V., Argiles A., Anderstam B., Brunet P. (2007). Review on uremic toxins III: Recommendations for handling uraemic retention solutes *in vitro*-towards a standardized approach for research on uraemia. Nephrol. Dial. Transplant..

[19] Schepers E., Glorieux G., Vanholder R. (2010). The gut: The forgotten organ in uremia?. Blood Purif..

[20] Evenpoel P., Meijers B.K., Bammens B.R., Verbeke K. (2009). Uremic toxins originating from colonic microbial metabolism. Kidney Int..

[21] Meijers B., Glorieux G., Poesen R., Bakker S.J. (2014). Nonextracorporeal methods for decreasing uremic solute concentration: A future way to go?. Semin. Nephrol..

[22] Jankowski J., Westhof T., Vaziri N.D., Ingrosso D., Perna A.F. (2014). Gases as uremic toxins: Is there something in the air?. Semin. Nephrol..

[23] Vanholder R., Seepers E., Pletinck A., Nagler E.V., Glorieux G. (2014). The uremic toxicity of indoxyl sulfate and *p*-cresyl sulfate: A systematic review. J. Am. Soc. Nephrol..

[24] De Loor H., Bammens B., Evenepoel P., de Preter V., Verbeke K. (2005). Gas chromatographic-mass spectrometric analysis for measurement of *p*-cresol and its conjugated metabolites in uremic and normal serum. Clin. Chem..

[25] Martinez A.W., Recht N.S., Hostetter T.H., Meyer T.W. (2005). Removal of *p*-cresol sulfate by hemodialysis. J. Am. Soc. Nephrol..

[26] Vanholder R., Bammens B., de Loor H., Glorieux G., Meijers B., Schepers E., Massy Z., Evenepoel P. (2011). Warning: The unfortunate end of *p*-cresol as a uraemic toxin. Nephrol. Dial. Transplant..

[27] Schiffer E., Mischak H., Vanholder R.C. (2008). Exploring the uremic toxins using proteomic technologies. Contrib. Nephrol..

[28] Langlois R.G., Trebes J.E., Dalmasso E.A., Ying Y., Davies R.W., Curzi M.P., Colston B.W., Turteltaub K.W., Perkins J., Chromy B.A. (2004). Serum protein profile alterations in hemodialysis patients. Am. J. Nephrol..

[29] Ward R.A., Brinkley K.A. (2004). A proteomic analysis of proteins removed by ultrafiltration during extracorporeal renal replacement therapy. Contrib. Nephrol..

[30] Kaiser T., Hermann A., Kielstein J.T., Wittke S., Bartel S., Krebs R., Hausadel F., Hillmann M., Golovko I., Koester P. (2003). Capillary electrophoresis coupled to mass spectrometry to establish polypeptide patterns in dialysis fluids. J. Chromatogr. A.

[31] Weissinger E.M., Kaiser T., Meert N., de Smet R., Walden M., Mischak H., Vanholder R.C. (2004). Proteomics: A novel tool to unravel the pathophysiology of uraemia. Nephrol. Dial. Transplant..

[32] Niwa T. (2011). Update of uremic toxin research by mass spectrometry. Mass Spectrom. Rev..

[33] Glorieux G., Mullen W., Duranton F., Filip S., Gayrard N., Husi H., Schepers E., Neirynck N., Schanstra J.P., Jankowski J. (2015). New insights in molecular mechanisms involved in chronic kidney disease using high-resolution plasma proteome analysis. Nephrol. Dial. Transplant..

[34] Miyata T., van Ypersele de Strihou C., Kurokawa K., Baynes J.W. (1999). Alterations in nonenzymatic biochemistry in uremia: Origin and significance of “carbonyl stress” in long-term uremic complications. Kidney Int..

[35] Galli F. (2007). Protein damage and inflammation in uraemia and dialysis patients. Nephrol. Dial. Transplant..

[36] Miyata T., Oda O., Inagi R., Iida Y., Araki N., Yamada N., Horiuchi S., Taniguchi N., Maeda K., Kinoshita T. (1993). β2-Microglobulin modified with advanced glycation end products is a major component of hemodialysis-associated amyloidosis. J. Clin. Investig..

[37] Pavone B., Bucci S., Sirolli V., Merlini G., del Boccio P., di Rienzo M., Felaco P., Amoroso L., Sacchetta P., Di Ilio C. (2011). β2-Microglobulin causes abnormal phosphatidylserine exposure in human red blood cells. Mol. Biosyst..

[38] Zwaal R.F., Comfurius P., Bevers E.M. (2005). Surface exposure of phosphatidylserine in pathological cells. Cell. Mol. Life Sci..

[39] Himmelfarb J., McMonagle E. (2001). Albumin is the major plasma protein target of oxidant stress in uremia. Kidney Int..

[40] Miyata T., Ueda Y., Yamada Y., Izuhara Y., Wada T., Jadoul M., Saito A., Kurokawa K., van Ypersele de Strihou C. (1998). Carbonyl stress in uremia: Accumulation of carbonyls accelerates the formation of pentosidine, an advanced glycation end product. J. Am. Soc. Nephrol..

[41] Pavone B., Sirolli V., Giardinelli A., Bucci S., Forlì F., di Cesare M., Sacchetta P., di Pietro N., Pandolfi A., Urbani A. (2011). Plasma protein carbonylation in chronic uremia. J. Nephrol..

[42] Mayer B., Zitta S., Greilberger J., Holzer H., Reibnegger G., Hermetter A., Oettl K. (2003). Effect of hemodialysis on the antioxidative properties of serum. Biochim. Biophys. Acta.

[43] Ward R.A., Ouseph R., McLeish K.R. (2003). Effects of high-flux hemodialysis on oxidant stress. Kidney Int..

[44] Pavone B., Sirolli V., Bucci S., Libardi F., Felaco P., Amoroso L., Sacchetta P., Urbani A., Bonomini M. (2010). Adsorption and carbonylation of plasma proteins by dialyser membrane material: *in vitro* and *in vivo* proteomics investigations. Blood Transfus..

[45] Miyata T., Saito A., Kurokawa K., van Ypersele de Strihou C. (2001). Advanced glycation and lipoxidation end products: Reactive carbonyl compounds-related uraemic toxicity. Nephrol. Dial. Transplant..

[46] Zoccali C., Mallamaci F., Tripepi G. (2000). AGEs and carbonyl stress: Potential pathogenetic factors of long-term uraemic complications. Nephrol. Dial. Transplant..

[47] Michelis R., Gery R., Sela S., Shurtz-Swirski R., Grinberg N., Snitkovski T., Shasha S.M., Kristal B. (2003). Carbonyl stress induced by intravenous iron during hemodialysis. Nephrol. Dial. Transplant..

[48] Kang J.H., Kim K.S., Choi S.Y., Know H.Y., Won M.H. (2001). Oxidative modification of human ceruloplasmin by peroxyl radicals. Biochim. Biophys. Acta.

[49] Miller Y.I., Altamentova S.M., Shaklai N. (1997). Oxidation of low-density lipoprotein by hemoglobin stem from a heme initiated globin radical: Antioxidant role of haptoglobin. Biochemistry.

[50] Rottoli P., Magri B., Cianti R., Bargagli E., Vagaggini C., Nikiforakis N., Pallini V., Bini L. (2005). Carbonylated proteins in bronchoalveolar lavage of patients with sarcoidosis, pulmonary fibrosis associated with systemic sclerosis and idiopathic pulmonary fibrosis. Proteomics.

[51] Lim P.S., Cheng Y.M., Yang S.M. (2007). Impairments of the biological properties of serum albumin in patients on hemodialysis. Nephrology.

[52] Massy Z.A., Borderie D., Nguyen-Khoa T., Drüeke T.B., Ekindjian O.G., Lacour B. (2003). Increased plasma *S*-nitrosothiol levels in chronic haemodialysis patients. Nephrol. Dial. Transplant..

[53] Weissinger E.M., Nguyen-Khoa T., Fumeron C., Saltiel C., Walden M., Kaiser T., Mischak H., Drüeke T.B., Lacour B., Massy Z.A. (2006). Effects of oral vitamin C supplementation in hemodialysis patients: A proteomic assessment. Proteomics.

[54] Badiou S., Cristol J.P., Jaussent I., Terrier N., Morena M., Maurice F., Leray-Moragues H., Rivory J.P., Chalabi L., Delcourt C. (2008). Fine-tuning of the prediction of mortality in hemodialysis patients by use of cytokine proteomic determination. Clin. J. Am. Soc. Nephrol..

[55] Mangé A., Goux A., Badiou S., Patrier L., Canaud B., Maudelonde T., Cristol J.P., Solassol J. (2012). HDL proteome in hemodialysis patients: A quantitative nanoflow liquid chromatography-tandem mass spectrometry approach. PLoS ONE.

[56] Weichhart T., Kopecky C., Kubicek M., Haidinger M., Döller D., Katholnig K., Suarna C., Eller P., Tölle M., Gerner C. (2012). Serum amyloid A in uremic HDL promotes inflammation. J. Am. Soc. Nephrol..

[57] Shao B., de Boer I., Tang C., Mayer P.S., Zelnick L., Afkarian M., Heineke J.W., Himmelfarb J. (2015). A cluster of proteins implicated in kidney disease is increased in high-density lipoprotein isolated from hemodialysis subjects. J. Proteome Res..

[58] Kopecky C., Haidinger M., Birner-Grünberger R., Darnhofer B., Kaltenecker C.C., Marsche G., Holzer M., Weichhart T., Antlanger M., Kovarik J.J. (2015). Restoration of renal function does not correct impairment of uremic HDL properties. J. Am. Soc. Nephrol..

[59] Ishikawa I., Chikazawa Y., Sato K., Nakagawa M., Imamura H., Hayama S., Yamaya H., Asaka M., Tomosugi N., Yokoyama H. (2006). Proteomic analysis of serum, outflow dialysate and adsorbed protein onto dialysis membranes (polysulfone and PMMA) during hemodialysis treatment using SELDI-TOF-MS. Am. J. Nephrol..

[60] Dihazi H., Muller C.A., Mattes H., Muller G.A. (2008). Proteomic analysis to improve adequacy of hemo- and peritoneal dialysis: Removal of small and highmolecular weight proteins with high- and low-flux filters or a peritoneal membrane. Proteomics Clin. Appl..

[61] Ficheux A., Gayrard N., Szware I., Andress D., Soullier S., Duny Y., Goubert G., Thomas M., Bismuth-Mondolfo J., Daurès J.P. (2011). The use of SDS-PAGE scanning of spent dialysate to assess uraemic toxin removal by dialysis. Nephrol. Dial. Transplant..

[62] Urbani A., Sirolli V., Lupisella S., Levi-Mortera S., Pavone B., Pieroni L., Amoroso L., di Vito R., Bucci S., Bernardini S. (2012). Proteomic investigations on the effect of different membrane materials on blood protein adsorption during hemodialysis. Blood Transfus..

[63] Pedrini L.A., Krisp C., Gmerek A., Wolters D.A. (2014). Patterns of proteins removed with high-flux membranes on high-volume hemodiafiltration detected with a multidimensional LC-MS/MS strategy. Blood Purif..

[64] Monari E., Cuoghi A., Bellei E., Bergamini S., Caiazzo M., Aucella F., Loschiavo C., Corazza L., Palladino G., Sereni L. (2014). Proteomic analysis of protein extraction during hemofiltration with on-line endogenous reinfusion (HFR) using different polysulphone membranes. J. Mater. Sci..

[65] Cuoghi A., Caiazzo M., Monari E., Bellei E., Bergamini S., Sereni L., Aucella F., Loschiavo C., Atti M., Tomasi A. (2015). New horizon in dialysis depuration: Characterization of a polysulfone membrane able to break the “albumin wall”. J. Biomater. Appl..

[66] Cornelis T., Eloot S., Vanholder R., Glorieux G., van der Sande F.M., Scheijen J.L., Leunissen K.M., Kooman J.P., Schalkwijk C.G. (2015). Protein-bound uraemic toxins, dicarbonyl stress and advanced glycation end products in conventional and extended haemodialysis and haemodiafiltration. Nephrol. Dial. Transplant..

[67] Chanard J., Lavaud S., Randoux C., Rieu P. (2003). New insights in dialysis membrane biocompatibility: Relevance of adsorption properties and heparin binding. Nephrol. Dial. Transplant..

[68] Pascual M., Tolkoff-Rubin N., Schifferli J.A. (1996). Is adsorption an important characteristic of dialysis membranes?. Kidney Int..

[69] Werner C., Jacobasch H. (1999). Surface characterization of polymers for medical devices. Int. J. Artif. Organs.

[70] Lonnemann G., Koch K.M., Shaldon S., Dinarello C.A. (1988). Studies on the ability of hemodialysis membranes to induce, bind, and clear human interleukin-1. J. Lab. Clin. Med..

[71] Anderson J.M., Bonfield T.L., Ziats N.P. (1990). Protein adsorption and cellular adhesion and activation on biomedical polymers. Int. J. Artif. Organs.

[72] Johnson R.J. (1994). Complement activation during extracorporeal therapy: Biochemistry, cell biology and clinical relevance. Nephrol. Dial. Transplant..

[73] Franck R.D., Weber J., Dresbach H., Thelen H., Weiss C., Floege J. (2001). Role of contact system activation in hemodialyzer-induced thrombogenicity. Kidney Int..

[74] Cheung A.K., Parker C., Wilcox L., Janatova J. (1990). Activation of complement by hemodialysis membranes: Polyacrylonitrile binds more C3a than cuprophan. Kidney Int..

[75] Pascual M., Schifferli J. (1993). Adsorption of complement factor D by polyacrylonitrile dialysis membranes. Kidney Int..

[76] Valette P., Thomas M., Dejardin P. (1999). Adsorption of low molecular weight proteins to hemodialysis membranes: Experimental results and simulations. Biomaterials.

[77] Vienken J. (2002). Polymers in nephrology. Characteristics and needs. Int. J. Artif. Organs.

[78] Bonomini M., Pavone B., Sirolli V., del Buono F., di Cesare M., del Boccio P., Amoroso L., di Ilio C., Sacchetta P., Federici G. (2006). Proteomics characterization of protein adsorption onto hemodialysis membranes. J. Proteom. Res..

[79] Urbani A., Lupisella S., Sirolli V., Bucci S., Amoroso L., Pavone B., Pieroni L., Sacchetta P., Bonomini M. (2012). Proteomic analysis of protein adsorption capacity of different hemodialysis membranes. Mol. Biosyst..

[80] Pieroni L., Levi Mortera S., Greco V., Sirolli V., Ronci M., Felaco P., Fucci G., De Fulviis S., Massoud R., Condò S. (2015). Biocompatibility of hemodialysis membrane materials by proteomic investigations. Mol. Biosyst..

[81] Aoike I. (2007). Clinical significance of protein adsorbable membranes-long-term clinical effects and analysis using proteomic technique. Nephrol. Dial. Transplant..

[82] Kato A., Takita T., Furuhashi M., Takahashi T., Watanabe T., Maruyama Y., Hishida A. (2001). Polymethylmethacrylate efficacy in reduction of renal itching in hemodialysis patients: Crossover study and role of tumor necrosis factor-α. Artif. Organs.

[83] Mares J., Thongbookerd V., Tumaz Z., Moravec J., Matejovic M. (2009). Specific adsorption of some complement activation proteins to polysulfone dialysis membranes during hemodialysis. Kidney Int..

[84] Ward R.A., Schafer R.M., Falkenhagen D., Joshua M.S., Heidland A., Klinkmann H., Gurland H.J. (1993). Biocompatibility of a new high-permeability modified cellulose membrane for haemodialysis. Nephrol. Dial. Transplant..

[85] Pesic I., Muller G.A., Baumann C., Dihazi G.H., Koziolek M.J., Eltoweissy M., Bramlage C., Asif A.R., Dihazi H. (2013). Cellulose membranes are more effective in holding back vital proteins and exhibit less interaction with plasma proteins during hemodialysis. Biochim. Biophys. Acta.

[86] Thongboonkerd V. (2010). Proteomics in extracorporeal blood purification and peritoneal dialysis. J. Proteomics.

